# Genome-Wide Expression Patterns of Rhoptry Kinases during the *Eimeria tenella* Life-Cycle

**DOI:** 10.3390/microorganisms9081621

**Published:** 2021-07-29

**Authors:** Adeline Ribeiro E Silva, Alix Sausset, Françoise I. Bussière, Fabrice Laurent, Sonia Lacroix-Lamandé, Anne Silvestre

**Affiliations:** Institut National de Recherche pour L’agriculture, L’alimentation et L’environnement (INRAE), Université de Tours, ISP, 37380 Nouzilly, France; adeline.ribeiro-e-silva@inrae.fr (A.R.E.S.); alix.sausset@inrae.fr (A.S.); francoise.bussiere@inrae.fr (F.I.B.); fabrice.laurent@inrae.fr (F.L.); sonia.lamande@inrae.fr (S.L.-L.)

**Keywords:** rhoptry kinases, gene expression, *Eimeria tenella*, life-cycle

## Abstract

Kinome from apicomplexan parasites is composed of eukaryotic protein kinases and Apicomplexa specific kinases, such as rhoptry kinases (ROPK). *Ropk* is a gene family that is known to play important roles in host–pathogen interaction in *Toxoplasma gondii* but is still poorly described in *Eimeria tenella*, the parasite responsible for avian coccidiosis worldwide. In the *E. tenella* genome, 28 *ropk* genes are predicted and could be classified as active (*n* = 7), inactive (incomplete catalytic triad, *n* = 12), and non-canonical kinases (active kinase with a modified catalytic triad, *n* = 9). We characterized the *ropk* gene expression patterns by real-time quantitative RT-PCR, normalized by parasite housekeeping genes, during the *E. tenella* life-cycle. Analyzed stages were: non-sporulated oocysts, sporulated oocysts, extracellular and intracellular sporozoites, immature and mature schizonts I, first- and second-generation merozoites, and gametes. Transcription of all those predicted *ropk* was confirmed. The mean intensity of transcription was higher in extracellular stages and 7–9 *ropk* were specifically transcribed in merozoites in comparison with sporozoites. Transcriptional profiles of intracellular stages were closely related to each other, suggesting a probable common role of ROPKs in hijacking signaling pathways and immune responses in infected cells. These results provide a solid basis for future functional analysis of ROPK from *E. tenella*.

## 1. Introduction

The Apicomplexa phylum consists of a large group of parasitic protists of medical and veterinary importance (*Plasmodium, Toxoplasma*, *Cryptosporidium*, *Eimeria*, etc). The genus *Eimeria* contains more than 1000 parasitic species, which invade the epithelial cell lining in the intestinal tract of their host, causing coccidiosis. All *Eimeria spp*. are considered to be highly host-specific. Avian coccidiosis is caused by seven *Eimeria* species, among which *Eimeria tenella, E. maxima*, and *E. acervulina* are the main species responsible for coccidiosis in poultry, worldwide. Clinical coccidiosis is associated with a reduction in body-weight gain, a decrease in the feed conversion index, and may result in a high mortality rate. Clinical and sub-clinical infections have a huge economic impact on poultry production. To control coccidiosis, coccidiostats are used as a feed additive in broilers production [1]. Attenuated live vaccines, which are very efficient, but expensive to produce, are mostly used in layers and labels with high economic importance in their production [2]. Natural alternative strategies, such as probiotics and phytochemicals, are becoming more explored [3].

**Figure 1:**
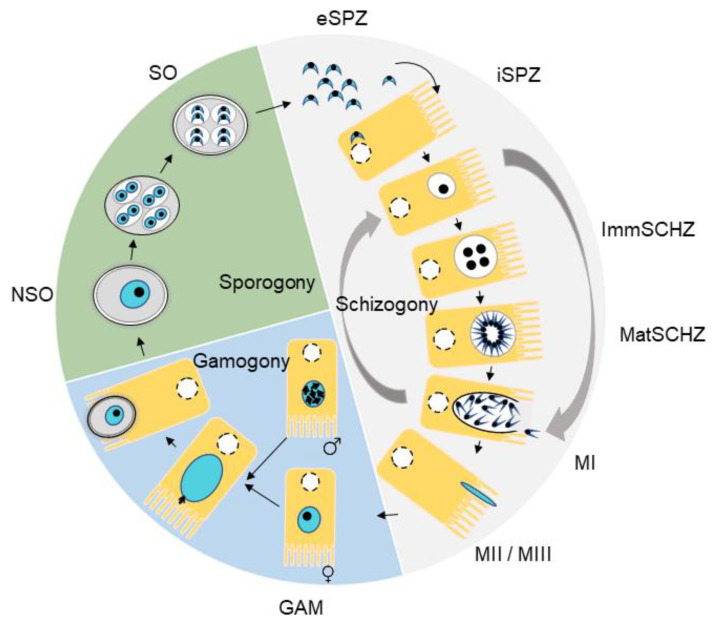
*Eimeria tenella* life-cycle. Sporogony occurs in the environment, whereas schizogony and gamogony occur in the chicken digestive tract. For the wild-type strain, three rounds of schizogony produce first-, second-, and third-generation merozoites. NSO, non-sporulated oocysts, SO sporulated oocysts, eSPZ extracellular sporozoites, iSPZ intracellular sporozoites, ImmSCHZ immature schizonts, MatSCHZ mature schizonts, MI first-generation merozoites, MII second-generation merozoites, and MIII third-generation merozoites.

The life-cycle of *Eimeria* is monoxenous with one phase in the environment and another in the host (asexual multiplication and sexual reproduction, respectively), with several developmental stages, either intracellular or extracellular (Figure 1), characterized by asynchronicity. Free-living stages consist of oocysts, which are very resistant to environmental conditions. After oral contamination of chickens with sporulated oocysts, released sporozoites invade intestinal epithelial cells and undergo three rounds of asexual multiplications (first-, second-, and third-schizogony), each generating a large number of merozoites. Third-generation merozoites differentiate into micro- and macro-gametes. Gamete fecundation results in the production of zygotes (gamogony). After maturation, zygotes evolve into non-sporulated oocysts that are released in the environment. In the environment, the sporogony will result in the production of sporulated oocysts under appropriate hygrometric and temperature conditions. The most pathogenic phase for the host is the merogony, during which successive intracellular multiplications induce digestive mucosal lesions. Live attenuated vaccines correspond to *Eimeria* strains that have only one merogony, leading to fewer mucosal lesions than wild-type (WT) strains.

The kinome of coccidia (*Toxoplasma, Neospora*, and *Eimeria*) is composed of eukaryotic protein kinases (ePKs) [4,5] and a coccidia-specific kinase family (rhoptry kinases, ROPK) [6]. ROPK sequences are highly divergent from ePK sequences [7] and include active kinases (i.e., a kinase domain organized in 12 subdomains that retain all the key residues needed for catalysis, as the catalytic triad KDD), pseudo-kinases (inactive enzymes, characterized by the absence of a complete catalytic domain and/or a mutated catalytic triad), and non-canonical kinases (i.e., active kinases with most of the residues necessary for catalysis, but with some differences in the conserved residues).

*Ropks* were amplified from an ancestral gene. *T. gondii* type I, II, and III contain 37, 55, and 38 *ropk*, respectively [6]. Individual *ropk* deletion in a type II *T. gondii* resulted in less virulent strains for 16 *ropk* genes [8]. Contrary to *Eimeria sp*., which develops in one host, *T. gondii* infects intermediate warm-blooded hosts for asexual multiplication (tachyzoite and bradyzoite stages) and reproduces sexually in cats as a definitive host (sporozoites and gametes). In *T. gondii*, ROPK is associated with host cell manipulation during asexual multiplication (i.e., tachyzoite stage): invasion process, virulence, and host cell modulation have been well documented ([9] for review). Although ROPK expression was also detected to a lesser extent, in merozoites stages [10,11], no clear function was identified due to the difficulty to purify these enteric stages ([12] for review).

This gene family remains mostly unknown in *E. tenella* [7,13]. Among 90 PKs predicted in the *E. tenella* genome, 28 are likely to belong to the ROPK family [7,14], among which, 18 *ropk* are organized in 6 tandem repeat loci. Within *E. tenella* ROPKs, 12 are predicted to be pseudo-kinases (i.e., inactive), 7 to be active kinases, and 9 to be non-canonical kinases (Table 1). *Ropk* genes from a tandem repeat locus belong to the same enzymatic class (i.e., active, inactive, or non-canonical kinases), except for the *Etrop5170/Etrop5190* locus for which the genes belong to inactive and active classes of kinase, respectively, and for the *Etrop28835/Etrop28855* locus which belongs to non-canonical and inactive kinases, respectively (see Table 1). Phylogenetic analysis showed that 19 ROPKs (i.e., 68%) from *E. tenella* were clustered into one specific clade [6]. This clade contains active (Eten3 sub-clade), inactive (Eten4, 5 and 6 sub-clades), and non-canonical kinases (Eten2a, 2b, and 3 sub-clades) (Figure S1). Several other *E. tenella* ROPKs were found outside this clade: either in one clade of unique ROPKs (6 ROPKs = 21%) or more closely related to *T. gondii* ROPK (*Tg*ROP21 and *Tg*ROP35) (3 ROPKs = 11%). *Ropk* genes from a tandem repeat locus are phylogenetically closer than other *ropk* genes (Figure S1). Only two ROPKs have been readily identified so far at the proteomic level in *E. tenella* sporozoites and four ROPKs at the merozoite stage [15]. In a previous study, the authors characterized the first active ROPK (*Et*ROP1) found in *E. tenella* sporozoites [16]: EtROP1 interacts and phosphorylates the cellular p53, resulting in the inhibition of host cell apoptosis and in the arrest of the host cell cycle in the G0/G1 phase. Here, we characterized *ropk* gene expression patterns during the *E. tenella* life-cycle, generating 9 developmental stages. Two WT strains, *Et*INRAE and *Et*WIS, from different geographical origins, were analyzed to describe the biological diversity of *ropk* transcription between strains. We confirmed the gene expression of those predicted *ropk* from the *E. tenella* genome in the various life stages. Their transcription in both extracellular and intracellular stages of *E. tenella* is consistent with their known functions in host-cell invasion and hijacking of host cell signaling pathways by *T. gondii*.

## 2. Materials and Methods

### 2.1. Ethics Statements

All animal experimentations have been performed in the Infectiology of Farm, Model, and Wildlife Animals Facility (PFIE, Centre INRAE Val De Loire: https://doi.org/10.15454/1.5572352821559333E12, accessed on 25 July 2021); member of the National Infrastructure EMERG’IN). Experimental protocols were designed in compliance with French law (2010/63/EU, 2010; Rural Code, 2018; Decree No. 2013-118, 2013) concerning the use of laboratory animals. Care and euthanasia of animals were practiced according to the national ethical guidelines and approved by the local ethics committee for animal experimentation (Comité d’Ethique en Expérimentation Animale Val de Loire, CEA VdL *N*°19): APAFIS#25884. The authors are committed to the principles of the 3Rs: reduction, refinement, and replacement of experimental animals.

### 2.2. Parasite Strains and Propagation

The Wisconsin (*Et*WIS) and INRAE (*Et*INRAE) strains of *E. tenella* were used throughout this study. *Et*WIS was first described in 1974 [17] and is a laboratory strain used in most studies. *Et*INRAE, initially described as a PAPt38 strain from INRA, was isolated from a poultry farm in France in 1974. The parasites were propagated by oral infection (10^4^ sporulated oocysts) of 3–5-week-old outbred PA12 chickens, reared in a coccidia-free environment with supply ad libitum of filtered water and anticoccidial- and antibiotic-free feed, following the standard protocol for oocyst purification [18].

### 2.3. Purification of Developmental Stages

We investigated nine distinct developmental stages of the parasites: non-sporulated oocysts (NSO), sporulated oocysts (SO), extracellular sporozoites (eSPZ), intracellular sporozoites (iSPZ), immature schizont (ImmSCHZ), mature schizont (MatSCHZ), first-generation merozoites (MI), second-generation merozoites (MII), and gametes (GAM). NSO were collected directly from the ceca at 7 days post-inoculation (p.i.) and purified as described previously [18]. SO were obtained after incubation in a solution of potassium dichromate 2.5% (*w*/*v*) for 48–72 h at 27 °C. Sporozoites were obtained from fresh fully sporulated oocysts. Briefly, after breaking the oocyst walls with 0.5 mm glass beads (Thermo Fisher Scientific), sporocysts were incubated in the excystation medium (0.25% (*w*/*v*) trypsin and 0.5% (*w*/*v*) biliary salts in 10 mM phosphate-buffered saline (PBS), pH 7.4) at 41 °C for 1 h. eSPZ were purified by a two-step filtration protocol, first on cotton and then on polycarbonate filters (pore size, 5 µm; Whatman). Second-generation merozoites and gametes were recovered from chicken caecal mucosa 112 h (5 d.p.i.) and 136 h (6 d.p.i.), after inoculation with 2.5 × 10^5^ or 1 × 10^4^ sporulated oocysts, respectively, as previously described [18,19]. Briefly, second-generation merozoites and gametes were purified from caecal content and scrapped infected mucosa at necropsies.

To generate the intracellular stages (i.e., iSPZ, ImmSCHZ, and MatSCHZ) and to cope with the asynchronicity of *E. tenella* development, we purified early stages from in vitro propagation. Freshly excysted, purified sporozoites were incubated with MDBK cells, at an MOI = 3 (5 × 10^5^ cells, 3 × 10^5^ cells and 2 × 10^5^ cells, respectively) in 6-well plates at 41 °C in 5% CO_2_. Infected cells were washed at 24 h p.i., for iSPZ stage, 48 h p.i., for ImmSCHZ stage, or at 62 h p.i., for MatSCHZ stage. For first-generation merozoites (MI) production, 2 × 10^6^ MDBK were seeded in a Petri dish and infected with 0.5–1 × 10^7^ freshly purified sporozoites. MI were recovered in the supernatant at 72 h p.i. and filtered on polycarbonate filters (pore size, 5 µm, Whatman).

### 2.4. RNA Extraction

Extraction was performed on 0.5–2 × 10^8^ parasites, whatever the stage. NSO and SO were resuspended in 50 µL of TRIzol and were fully disrupted by vortexing with 0.5 mm glass beads (Thermo Fisher Scientific, Waltham, MA, USA) for 1 min followed by cooling for 1 min in ice; this procedure was repeated 5 times. The lysate was then added to 950 µL of TRIzol provided in the kit (Direct-zol RNA Microprep, Zymo Research Corporation, Irvine, CA, USA), and the RNA extraction was performed according to the manufacturer’s protocol. For other developmental stages, parasites were directly lysed with TRIzol and RNA extraction was performed following the standard protocol of the kit. RNA concentration was measured using a NanoDrop One spectrophotometer (Thermo Fisher Scientific, Waltham, MA, USA). RNA integrity was checked according to an RNA Integrity Number (RIN > 8) using RNA 6000 Nano chips run on a Bioanalyzer 2100 (Agilent Technologies, Santa Clara, CA, USA). The samples were stored at −80 °C until further use. All samples were tested for genomic contamination using a negative control, which consisted of RNA extraction and PCR amplification without adding the RT enzyme.

cDNA was synthesized using the M-MLV Reverse Transcriptase (Promega Corporation, Madison, WI, USA), with random hexamer primers and oligo (dT)15 primer (Promega). cDNAs were then amplified by qPCR using the SYBR Green master mix (Bio-Rad Corporation, Hercules, CA, USA). Housekeeping genes used to normalize *ropk* gene amplification were: *Et18s* (EF210326) and *Etactin* (ETH_00009555). Stage-specific gene expression was determined using *Etama1* (ETH_00007745) for the sporozoite stage, *Etsag3* (ETH_00010755) for the schizont, and merozoite stages, and *Etgam56* (ETH_00007320) for macrogametes [20] (Figure S2). All ROPK and host-cell-specific primers are listed in (Table S1).

The protocol used for qPCR was: 95 °C for 5 min and 40 cycles at 95 °C for 10 s and 60 °C (or 62 °C) for 15 s. The absence of primer dimers in the reaction was verified with melting curves, performed at 60 °C (or 62 °C) for 5 s followed by gradual heating (0.5 °C/s) to 95 °C. The absence of cross-reactivity of parasite primers with host cell nucleic acid (and host cell primers with parasite nucleic acid) was verified (data not shown). qPCRs were performed in triplicate for each experiment. ROPK and stage-specific gene expression were normalized to Ct values obtained for *Et18s* and *Etactin* using the formula: 2^−(Ct *Et* specific stage gene or ropk–Ct mean of *Et* housekeeping genes)^. Gametes, being the stage with the lowest *ropk* transcription level (present study and [18,19]), gene expression values were standardized to gamete stage expression: genes with a fold change (FC) in expression > 2 were considered as expressed genes at that particular stage. Gene expression values are expressed as the mean ± SD of two independent biological replicates.

### 2.5. Gene Expression Profiling and Hierarchical Clustering

To examine the global expression profiles of ROP kinase genes among different developmental stages, a principal component analysis (PCA) and a hierarchical clustering analysis (HCA) with average linkage (Pearson distance) were carried out using the R software [21]. The variables located near the origin of the axis were of little value in interpreting the factorial plan of projection, whereas variables found on the circle were highly significant in describing the data. The proximity of variables (and/or data) on a plan showed that they were positively correlated. The interest of each plan of projection was assessed by its inertia (percentage of variance). The regressions were established by the least-squares method. The heatmap was illustrated with Heatmapper software [22].

## 3. Results

**Figure 2:**
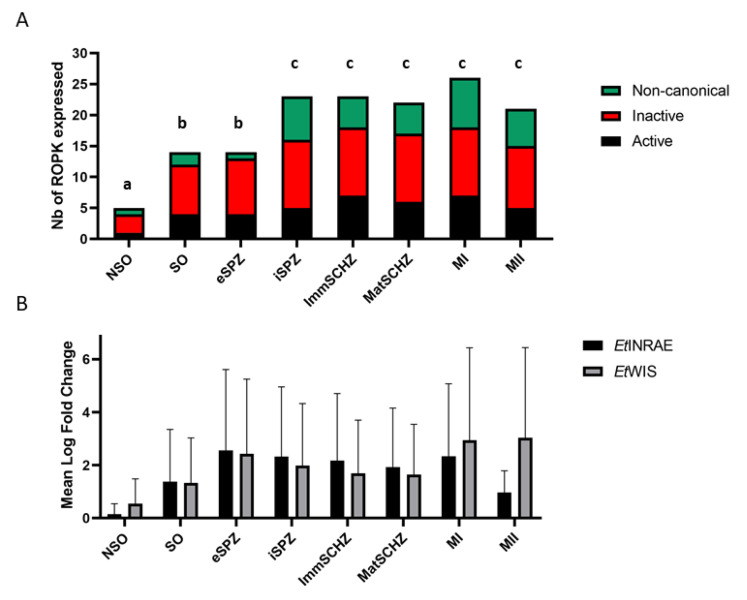
(**A**) The number of rhoptry kinases ROPK expressed in each developmental stage for *Et*INRAE, according to the putative classes of kinase activity (non-canonical in green, inactive in red, and active in black). Gene expression was standardized with the expression level in gametes: genes with a fold change > 2 were considered as transcribed. Two-way ANOVA, different letters (a, b, c) refer to statistical differences between the groups (*p* < 0.05). “a” means that the number of ROPK expressed is statistically different between NSO and all other stages, “b” means that the number of ROPK expressed is not statistically different between SO and eSPZ stages, but it is statistically different in comparison with all other stages, “c” means that the number of ROPK expressed is not statistically different between iSPZ, ImmSCHZ, MatSCHZ, MI, and MII stages, but it is statistically different in comparison with NSO, SO, and eSPZ. (**B**) The expression level of *ropk* was quantified in each developmental stage for *Et*INRAE (black) and *Et*WIS (grey) strains, expressed as log fold change (FC) in comparison with the gamete stages. Data are expressed as the mean ± SD of two independent experiments. Two-way ANOVA showed no significant difference between strains and between developmental stages.

*Ropk* transcription is regulated across the *E. tenella* life-cycle in the *Et*INRAE strain (Figure 2A and Table S2). A significant and gradual increase in the number of transcribed *ropk* is observed during the parasite cycle. *Ropk* gene expression is the lowest in free-living stages and gradually increases with sporulation, and even more in the intracellular stages (schizonts and merozoites). The transcription of all three classes of ROPK is similar, independently of the enzymatic status of the kinase (active, inactive, and non-canonical). The level of expression, expressed as log of fold change compared to the gamete stage expression, is the highest in extracellular sporozoites and MI stages (Figure 2B). Although the number of transcribed *ropk* is not significantly different between SO and eSPZ, the intensity of transcription is 10-fold higher in eSPZ. Those results were confirmed with the *Et*WIS strain (Figure 2B and Supplementary data, Figure S3) except for the MII stage in which *ropk* transcription levels tended to be lower in the *Et*INRAE strain in comparison with *Et*WIS. As the quality of mRNA samples was checked, the lower transcription of *ropk* in the *Et*INRAE MII stage may be explained by biological diversity.

**Figure 3:**
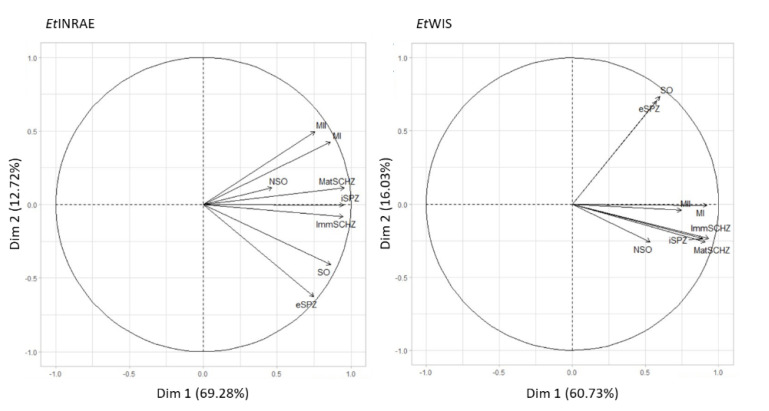
Principal component analysis (PCA) of rhoptry kinase transcription at different stages of development of the *Eimeria tenella* life-cycle. NSO non-sporulated oocysts, SO sporulated oocysts, eSPZ extracellular sporozoites, iSPZ intracellular sporozoites, ImmSCHZ immature schizont, MatSCHZ mature schizont, MI first-generation merozoites, and MII second-generation merozoites.

A strong and positive correlation was observed between the transcriptional profile of three groups of developmental stages in *Et*INRAE (Figure 3): (i) the three intracellular stages (iSPZ, ImmSCHZ, and MatSCHZ) were closely related to each other; whereas, (ii) SO showed an expression pattern similar to eSPZ, and (iii) MI and MII profiles were highly correlated. These correlations are consistent with the nature and the similarity of the developmental stages. In the *Et*WIS strain, similar correlations were confirmed, except for the merozoite profiles that were more closely related to the intracellular stage profiles than the ones observed in the *Et*INRAE strain (Figure 3).

**Figure 4:**
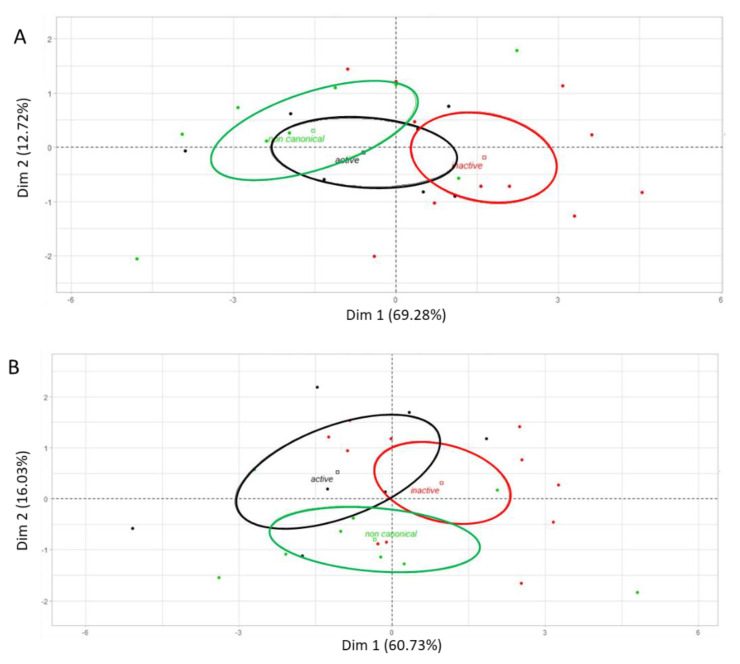
Principal component analysis of rhoptry kinase transcription according to their activity status in *Et*INRAE (**A**) and *Et*WIS (**B**) strains. Active kinases are represented in black, inactive in red, and non-canonical in green.

The PCA supports a wide distribution of ROPKs, with a partial overlap of the active kinases with either non-canonical or inactive kinases (Figure 4A). Although active and non-canonical kinase groups were partly differentially represented in the *Et*WIS strain (Figure 4B), no significant differences were observed between global kinase transcription for both strains (Figure S4).

**Figure 5:**
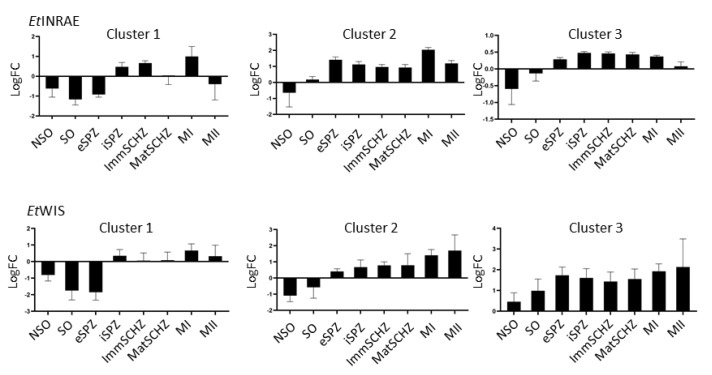
Expression profile clustering of the rhoptry kinases in different stages of development of *Eimeria tenella*. Histograms show log fold change (Log FC) in the stage of development vs. gamete stage; *Etactin* and *Et18s* served as housekeeping genes.

The hierarchical clustering analysis, based on the PCA, showed three clusters according to the stages in which they are transcribed across the life-cycle of *E. tenella* but no sub-clustering based on ROPKs activity (Figure 5 and Table S2). This is illustrated with the paragon of each cluster (i.e., the nearest ROPK of each cluster barycentre). In the *Et*INRAE strain, (i) cluster 1 contained *ropks* that were transcribed neither in oocysts (non-sporulated and sporulated) nor in eSPZ; (ii) cluster 2 presented *ropk*s that were not transcribed in non-sporulated oocysts only, which included the large majority of ROPKs; and finally, (iii) cluster 3 was composed of *ropks* that were not transcribed in oocysts only (non-sporulated and sporulated). In the *Et*WIS strain, 3 clusters were also found according to the stages in which *ropks* were transcribed. Cluster 1 was similarly defined between strains, but clusters 2 and 3 slightly differed between the strains.

**Figure 6:**
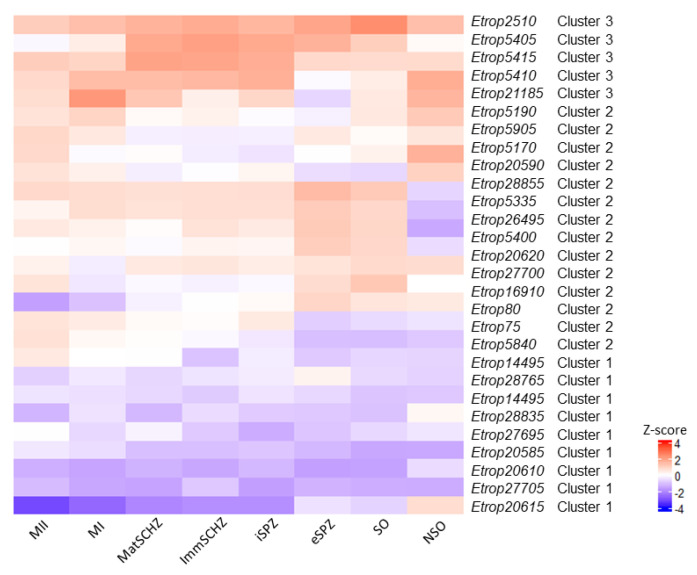
Heatmap of differentially transcribed rhoptry kinase genes in the *Et*INRAE strain. Red color indicates a rhoptry kinase transcription level higher than the mean, and blue color represents a transcription level lower than the mean. NSO non-sporulated oocysts, SO sporulated oocysts, eSPZ extracellular sporozoites, iSPZ intracellular sporozoites, ImmSCHZ immature schizonts, MatSCHZ mature schizonts, MI first-generation merozoites, and MII second-generation merozoites. The cluster resulting from the hierarchical clustering analysis are indicated.

**Figure 7:**
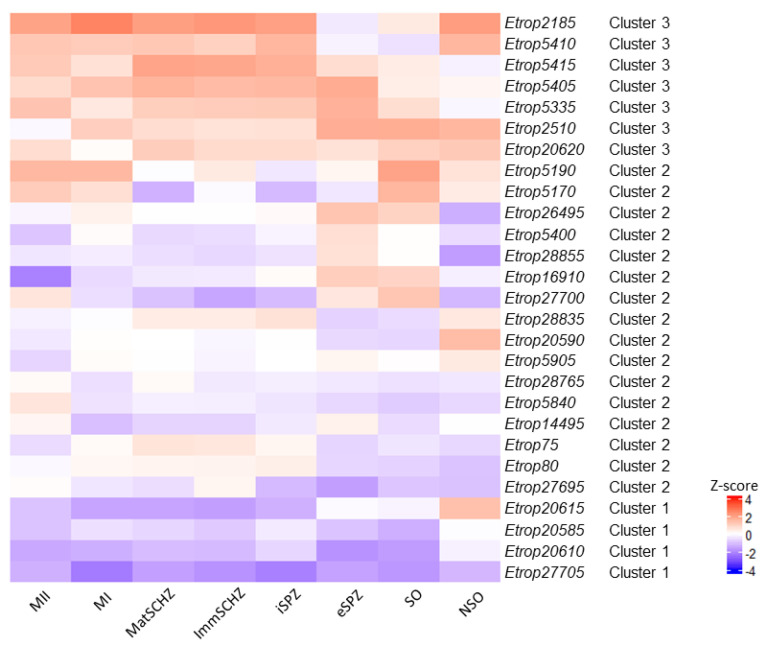
Heatmap of differentially transcribed rhoptry kinase genes in the *Et*WIS strain. Red color indicates a rhoptry kinase transcription level higher than the mean and blue colour represents a transcription level lower than the mean. NSO non-sporulated oocysts, SO sporulated oocysts, eSPZ extracellular sporozoites, iSPZ intracellular sporozoites, ImmSCHZ immature schizonts, MatSCHZ mature schizonts, MI first-generation merozoites, and MII second-generation merozoites. The cluster resulting from the hierarchical clustering analysis are indicated.

A heatmap representation of the transcription profiles for *ropk* genes for the *Et*INRAE and *Et*WIS strains are shown in Figure 6 and Figure 7, respectively. The clustering of *ropks* is consistent with the profiles of expression, with a lower transcription of cluster 1 ropks in early stages, a higher transcription of *ropks* from cluster 3 in intracellular stages, and cluster 2 appears less homogeneous. The heatmap also illustrates the diversity of *ropks* transcription inside each cluster: a slight difference may be observed between *ropks* from a cluster and the *ropk* paragon. The z-score for a given *ropk* corresponds to this *ropk* transcription in comparison to all other *ropk* transcription in this particular stage. So, a given *ropk* can be described as “overexpressed” (in comparison to the GAM stage, in Figure 5) but it may be “less overexpressed” than the mean of all *ropk*, and consequently, be illustrated in blue color in the heatmap (Figure 6 and Figure 7). The increase in *ropk* transcription during sporulation is clearly shown, and a higher transcription of *ropk* in intracellular stages is obvious.

**Figure 8:**
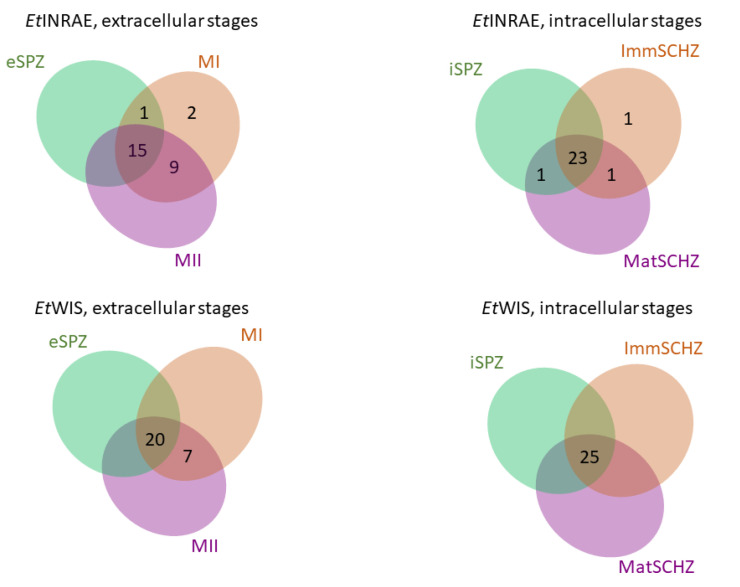
Venn diagram of differentially transcribed rhoptry kinases in three extracellular stages and three intracellular stages of the *Et*INRAE and *Et*WIS strains. eSPZ extracellular sporozoites, iSPZ intracellular sporozoites, ImmSCHZ immature schizonts, MatSCHZ mature schizonts, MI first-ge-neration merozoites, and MII second-generation merozoites. In extracellular stages, 27 *ropks* were transcribed, in intracellular stages, 26 and 25 *ropks* were transcribed in *Et*INRAE and *Et*WIS, respectively.

*Ropks* that were differentially transcribed between parasitic stages (sporozoites, schizonts, and merozoites) were combined in a Venn diagram to identify *ropks* related either to invasive stages or to intracellular stages (Figure 8 and Table S2). *Ropks* profiles of intracellular stages were highly similar between strains: the same 23 for *Et*INRAE and 25 out of 26 *ropks* for *Et*WIS (*Etrop20615* being not transcribed in those stages) were expressed in iSPZ, ImmSCHZ, and MatSCHZ. More differences were observed between extracellular stages: eSPZ, MI, and MII share the transcription of 15 and 20 out of 27 *ropks*, in *Et*INRAE and *Et*WIS, respectively. The 9 *ropk* transcribed in both MI and MII, but not in eSPZ, in the *Et*INRAE strain are partly the same as the 7 *ropk* in the *Et*WIS strain: *Etrop75, Etrop80, Etrop20585, Etrop20590*, and *Etrop28835* are common to both strains; *Etrop5840, Etrop21185, Etrop27695*, and *Etrop28765* are shared by *Et*INRAE MI and MII stages, and *Etrop20610* and *Etrop27705* are shared by *Et*WIS MI and MII stages. No *ropks* were found specific to the MII stage: *ropks* expressed in MII are either also expressed in eSPZ or in MI. The future characterization of those ROPK would be very interesting to decipher, including how different developmental extracellular stages invade and hijack caecal epithelial cells.

## 4. Discussion

Protein kinases are key regulators of host–pathogen interactions. In particular, the ROP kinase family, mostly described in *T. gondii*, is required for survival and development throughout the parasite life-cycle. We carried out a comprehensive transcriptional analysis of the *ropk* gene family, in *E. tenella* (free-living stages, extracellular and intracellular stages). As observed in *T. gondii*, where the *ropk* genes share a similar expression profile [23,24], our data reveal a dynamic and regulated transcription of *ropk* genes, along the *E. tenella* life-cycle. We confirmed a global increase in *ropk* transcription during the sporulation, as described for *Plasmodium berghei* [25]. We also noticed a stronger *ropk* gene expression in the intracellular stages of the parasite development (iSPZ, ImmSCHZ, and MatSCHZ), which might be associated with known functions of ROPK on cell invasion and intracellular development of *T. gondii* [26,27]. The co-transcription of active ROPK with either inactive or non-canonical kinases is consistent with the complex co-expression of ROPK observed in *T. gondii.* The inactive kinase *Tg*ROP5 is a co-factor of active ROPK, regulating *Tg*ROP17 and *Tg*ROP18 activity [28,29,30,31]. In *E. tenella*, the inhibition of host cell apoptosis by *Et*ROP1 is independent of *Et*ROP1 kinase activity [16], suggesting the involvement of at least another active kinase.

A very strong correlation was observed between *ropk* gene transcription from iSPZ, ImmSCHZ, and MatSCHZ. As sporozoites do not engage their development in a synchronous manner, we cannot rule out the possible presence of few intracellular sporozoites in immature schizonts samples. A similar situation may also occur for immature schizonts in mature schizonts samples. To the best of our knowledge, no specific gene has been identified to confirm the absence of developmental stages contamination for these three successive stages. However, *Etrop20610*, which is transcribed in intracellular sporozoites of the *Et*INRAE strain, was not detected in immature schizonts. Moreover, the similarity of transcriptional profiles of successive developmental stages was also observed between sporulated oocysts and extracellular sporozoites, through genome-wide transcriptomic analysis (not only the *ropk* gene family) [32]. For MI and MII, the similarity of *ropk* expression profiles (also confirmed by [32]) cannot be explained by any contamination, as MI were recovered from the supernatant of cultured cells (in vitro) whereas MII were purified from infected caecal cells (in vivo). Taken together, these results support the fact that even if minor contamination exists between intracellular stages, the *ropk* gene transcription patterns observed across the developmental stages of *E. tenella* are correct and reliable.

*Tg*ROPK are broadly known to be involved in host cell invasion and hijacking of host signaling pathways and immune responses in *T. gondii*. Venn diagram allows identification of candidate genes involved into extracellular and intracellular stages in *E. tenella*. The 9 *ropk* transcribed in MI and MII but not in eSPZ in the *Et*INRAE strain are partly the same as the 7 *ropk* in the *Et*WIS strain. These data are consistent with the proteomic data identifying merozoites specific ROPK expression [15]. The future functional characterization of those ROPK would be very useful to decipher how different developmental extracellular stages invade and hijack caecal epithelial cells. Concerning intracellular stages, *ropk* gene transcription was highly similar and no transcriptional signature could be associated with the extracellular or intracellular stages of *E. tenella* in both strains. To date, only *Et*ROP5190 [16] has been functionally characterized and associated with cell apoptosis inhibition, which is consistent with *Etrop5190* transcription in iSPZ (sporozoites early invading epithelial cell) and ImmSCHZ, and MatSCHZ.

Although the main part of the *ropk* family is transcriptionally regulated, some differences may be observed at the protein level. Indeed, post-transcriptional modifications and translational control are frequent in Apicomplexa (for a recent review [33]). Proteomic data on *E. tenella* are scarce. To date, no exhaustive proteomic analysis of the ROPK library has been published on the developmental stages analyzed in the present study to support our analysis. However, some proteomic data are available on sporozoites [15,34,35,36,37] and merozoites [36,38,39]. Previous publications demonstrated that the higher transcription of *Etrop75*, *Etrop5400*, *Etrop5840*, *Etrop5905*, *Etrop26495*, *Etrop27695*, and *Etrop28765* observed in merozoites in the present study is confirmed at the protein level [36,38], and the higher transcription of *Etrop27700* in sporozoites is consistent with proteomic data [15].

Studying two different wild-type *E. tenella* strains, the results showed a remarkable congruence. The main difference between strains was the greater similarity of MI and MII transcriptional patterns with intracellular stages patterns in the *Et*WIS strain compared to the *Et*INRAE strain. Although non-significant, the mean transcription level of *ropk* tends to be higher in *Et*WIS MII than in *Et*INRAE MII. Although both strains have an equivalent virulence status (data not shown), *Et*INRAE was propagated on PA12 White Leghorn chickens since its isolation from the field, whereas *Et*WIS was recently obtained from the Royal Veterinary College (London, UK), where it was propagated on Light Sussex, White Leghorn or Cobb500 chickens. This difference may account for a local adaptation of the *Et*INRAE strain. It is noteworthy that the difference in *ropks* expression between *Et*INRAE and *Et*WIS resides mainly in *ropks* expression in the extracellular stages. We may speculate that co-evolution acted more on the host-cell invasion step than on intracellular parasite development. To determine whether this strain specificity of *ropk* transcription may impact the virulence or fitness of *E. tenella*, supplementary experiments would be necessary. Some loci mapped to chromosome 2 have been associated with precociousness in *E. tenella* [40], and transcriptomic differences were found between virulent and precocious strains [41]. The comparison of *ropk* transcription between WT and attenuated (or precocious) strains may be very useful to identify new stage-specific ROPKs, possibly involved in virulence.

## 5. Conclusions

A comprehensive expression analysis of the *ropk* gene family in *Eimeria tenella* was conducted for the first time, all along the life-cycle (free-living, extracellular, and intracellular stages). If ROPK protein expression confirms the transcriptional data, a large number of ROPK could be co-expressed, suggesting some complex interactions between active and inactive, or non-canonical ROPK, as observed in *T. gondii.* Our results suggest that *ropks* are a large family of multi-functional genes which are expected to play essential roles in parasite development and hijacking of host cell signaling pathways. This exhaustive inventory of poorly characterized and developmentally regulated *ropk* genes provides a solid basis for future functional studies of the ROPKs in *E. tenella*, (as published for *Etrop5190* [16]) and paves the way for the development of new strategies/vaccines to control avian coccidiosis.

## Figures and Tables

**Table 1:** Rhoptry kinase genes from the *Eimeria tenella* genome.

Activity	Gene ID	Gene Symbol	Phylogeny ^1^
active	ETH_00005190	*Etrop5190*	ROPK unique
active	ETH_00005905	*Etrop5905*	*Tg*ROP35
active	ETH_00014495	*Etrop14495*	*Tg*ROP21
active	ETH_00026495	*Etrop26495*	*Tg*ROP35
active	ETH_00027695	*Etrop27695*	Eten1
active	ETH_00027700	*Etrop27700*	Eten1
active	ETH_00027705	*Etrop27705*	Eten1
inactive	ETH_00000075	*Etrop75*	Eten4
inactive	ETH_00000080	*Etrop80*	Eten4
inactive	ETH_00002510	*Etrop2510*	Eten6
inactive	ETH_00005170	*Etrop5170*	ROPK unique
inactive	ETH_00005335	*Etrop5335*	ROPK unique
inactive	ETH_00005400	*Etrop5400*	Eten5
inactive	ETH_00005405	*Etrop5405*	Eten5
inactive	ETH_00005410	*Etrop5410*	Eten5
inactive	ETH_00005415	*Etrop5415*	Eten5
inactive	ETH_00016910	*Etrop16910*	ROPK unique
inactive	ETH_00028835	*Etrop28835*	ROPK unique
non-canonical	ETH_00005840	*Etrop5840*	Eten3
non-canonical	ETH_00020585	*Etrop20585*	Eten3
non-canonical	ETH_00020590	*Etrop20590*	Eten3
non-canonical	ETH_00020610	*Etrop20610*	Eten3
non-canonical	ETH_00020615	*Etrop20615*	Eten3
non-canonical	ETH_00020620	*Etrop20620*	Eten3
non-canonical	ETH_00021185	*Etrop21185*	Eten3
non-canonical	ETH_00028765	*Etrop28765*	Eten2a
non-canonical	ETH_00028855	*Etrop28855*	Eten2b

^1^ Adapted from [6]. See text for details about the phylogeny and supplementary data Figure S1.

## Data Availability

All raw data are available as Supplementary Materials Table S2.

## Supplementary Materials

The following are available online at https://www.mdpi.com/article/10.3390/microorganisms9081621/s1. Table S1: PCR primers ROPK and stage-specific primers. Table S2: Mean *ropks* transcription in developmental stages. Fold change *ropks* transcription levels in *Et*INRAE and *Et*WIS strains, in comparison to gamete stage. Differential *ropks* expressed in extracellular and intracellular stages, in *Et*INRAE and *Et*WIS strains. Figure S1: Phylogeny of rhoptry kinases (ROPK) from *Eimeria tenella*. ROPK amino acid sequences from *E. tenella* (Houghton strain) were recovered from VEuPathDB (release 52, 20 May 2021). Phylogeny software (Muscle program for alignment and maximum-likelihood method) was used [42]. The number at each node represents the percentage bootstrap support, calculated from 1,000 replicates. Figure S2: Stage-specific genes transcription during *E. tenella* development (*Et*INRAE (A) and *Et*WIS (B) strains). *Et18s* (EF210326) and *Etactin* (ETH_00009555) were used as housekeeping genes. Stage-specific genes were *Etama1* (ETH_00007745) for sporozoites, *Etsag3* (ETH_00010755) for schizonts and merozoites and *Etgam56* (ETH_00007320) for macrogametes [20]. Data are expressed as the mean ± SD of two independent experiments. Figure S3: Number of rhoptry kinases expressed in each stage of *E. tenella* development (EtWIS), according to the kinase activity (non-canonical, inactive, and active). Gene expression was standardized with the expression level in gametes: genes with a fold change > 2 were considered as transcribed. Two-way ANOVA, different letters refer to statistical differences (*p* < 0.05). Figure S4: Principal component analysis of rhoptry kinases expression according to the *Eimeria tenella* strain. *ropk* in *Et*INRAE are represented in black and in *Et*WIS in red.
