# Supplementary material for: Genome-Wide Expression Patterns of Rhoptry Kinases during the Eimeria tenella Life-Cycle
Source: Microorganisms. 2021 Jul 29;9(8):1621. doi: 10.3390/microorganisms9081621 (PMC8399136; doi:10.3390/microorganisms9081621)
Supplement: Supplementary file 1 [file microorganisms-09-01621-s001.zip › Table-S1-revised.pdf]

Table S1. PCR primers ROPK and stage specific primers.

Accession No.	Gene Symbol		Primer sequence (5'-3')	Title 1	Title 2	Title 3
ETH_00000075	*Etrop75*	F	GACTCTTGGTGCCTCTGTTTAC	entry 1	data	data
		R	TCTCCCACGAGCTCCTCATA	entry 2	data	data [1]
ETH_00000080	*Etrop80*	F	TACGGTGCTTGCCCTAACAG			
		R	CTGTCCCTTGTGCTGGTCTT			
ETH_00002510	*Etrop2510*	F	GCTGGGAATGACAATTCGGC			
		R	CCCTCGGTTTCTCGATCCTG			
ETH_00005170	*Etrop5170*	F	GGCCAAGTGTTCCTTAACGC			
		R	CGACTCTCACTGCTTGCTGA			
ETH_00005190	*Etrop5190*	F	GGTCGCAAAGACTGCCAAGA			
		R	CCATAGGGCGTCTGAACTGT			
ETH_00005335	*Etrop5335*	F	CCTTTGGAGGCGAAGTGCTA			
		R	GTCAGCCCTCATGAAGCAGT			
ETH_00005400	*Etrop5400*	F	CACCTGTGAAGACGCTGAAC			
		R	GCAGATCCATAATCAACGACCG			
ETH_00005405	*Etrop5405*	F	GGGCTTGGGTGTTTGTCTATAC			
		R	CCTTCATAAACGAGAAGACGGATG			
ETH_00005410	*Etrop5410*	F	GGAGTAAGTTGGCAGTCGCT			
		R	GCATCCAGTCTCTCGTCTCTG			
ETH_00005415	*Etrop5415*	F	TATGGACTGGGCGGTGACTT			
		R	GCGGATACTCTTGCCGATGT			
ETH_00005840	*Etrop5840*	F	GCAAACTCCTCATGTTTCCCC			
		R	CGAGTAAAACTGGCGAAGAGC			
ETH_00005905	*Etrop5905*	F	ACGGCGAGTACCAAGTCTTC			
		R	GTCCGTCATGTGGCGAATCA			
ETH_00014495	*Etrop14495*	F	CCTCAGCAGCGAAGCAATAG			
		R	GGTTTGTGGGAGAAAAGAGGC			
ETH_00016910	*Etrop16910*	F	ACAGCAGCACTGGAGAAACT			
		R	CTTGAAGAGTCCAGCGGTCT			
ETH_00020585	*Etrop20585*	F	AAAGGTCCGTTCAGATGTGCT			
		R	TGCGTGAGGTCTGGGAACT			
ETH_00020590	*Etrop20590*	F	GTGGGGAACGGAGACGAAAT			
		R	CCAAACACCGCAAGATGAGC			
ETH_00020610	*Etrop20610*	F	CAAGACCCGGACTGAAACGA			
		R	GGGAAGTTACCAAGACGGGG			
ETH_00020615	*Etrop20615*	F	ACCTGGACAGAACGGAGAAT			
		R	CTCTAATAGCCGCAGAATCACC			
ETH_00020620	*Etrop20620*	F	GAAGGGAGCACATTTGGGGA			
		R	CCGAAATGGCACGTTGTGAG			
ETH_00021185	*Etrop21185*	F	GTGGTACGAAATGGAGGACTC			
		R	CCTGTTGACTCTTTCGGGC			
ETH_00026495	*Etrop26495*	F	GGCTTCCTGCACAGAGACTT			
		R	CTTGGGCGAAAAAGGCACAA			
ETH_00027695	*Etrop27695*	F	CGTTTGTTGGAGCCGTCATC			
		R	ATAGCACCTGTTCGCCTCAG			
ETH_00027700	*Etrop27700*	F	CTAAAGGAGCACGGGGTGTC			
		R	ACGGGAAAGTGGGGGTACTT			
ETH_00027705	*Etrop27705*	F	TTGCTGACGTGGTATTGCAG			
		R	CGGCTTTCGTAGGTAGTTCG			
ETH_00028765	*Etrop28765*	F	CATTGGCCGCCGTATTATGC			
		R	CTTCTTTGCCCACATTCCGC			
ETH_00028835	*Etrop28835*	F	GCGTCTAGAATCCGATACGAC			
		R	CCATCTCAGTGGCACTTCAG			
ETH_00028855	*Etrop28855*	F	GATCAAGCAAGCAGGTTAACGTC			
		R	GCCAAAAGCAATTCAGAAAGCGC			

ETH_00007745	*Etama1*	F	TGGGGAACGACAAAGAGGTG
		R	TCCGAATCCAGGGGCTGATA
ETH_00010755	*Etsag3*	F	CCAGACAAAGGGCTCTCAACA
		R	CAATCTTCCGCCACACCTCT
ETH_00007320	*Etgam56*	F	GACGGAGCCAACGAACTGAT
		R	ATGGGGGTGGTCTCTTCCTT
EF210326	*Et18s (E. tenella)*	F	CTATGGGTGGTGGTGCATGG
		R	CCGCAGAGTTACCGACCCTA
ETH_00009555	*Etactin (E. tenella)*	F	GAAGAGATGAAGAATGCTGAGG
		R	GATGGATACCCGATGCCTC
