# Supplementary figures and images for: Genome-Wide Expression Patterns of Rhoptry Kinases during the Eimeria tenella Life-Cycle
Source: Microorganisms. 2021 Jul 29;9(8):1621. doi: 10.3390/microorganisms9081621 (PMC8399136; doi:10.3390/microorganisms9081621)

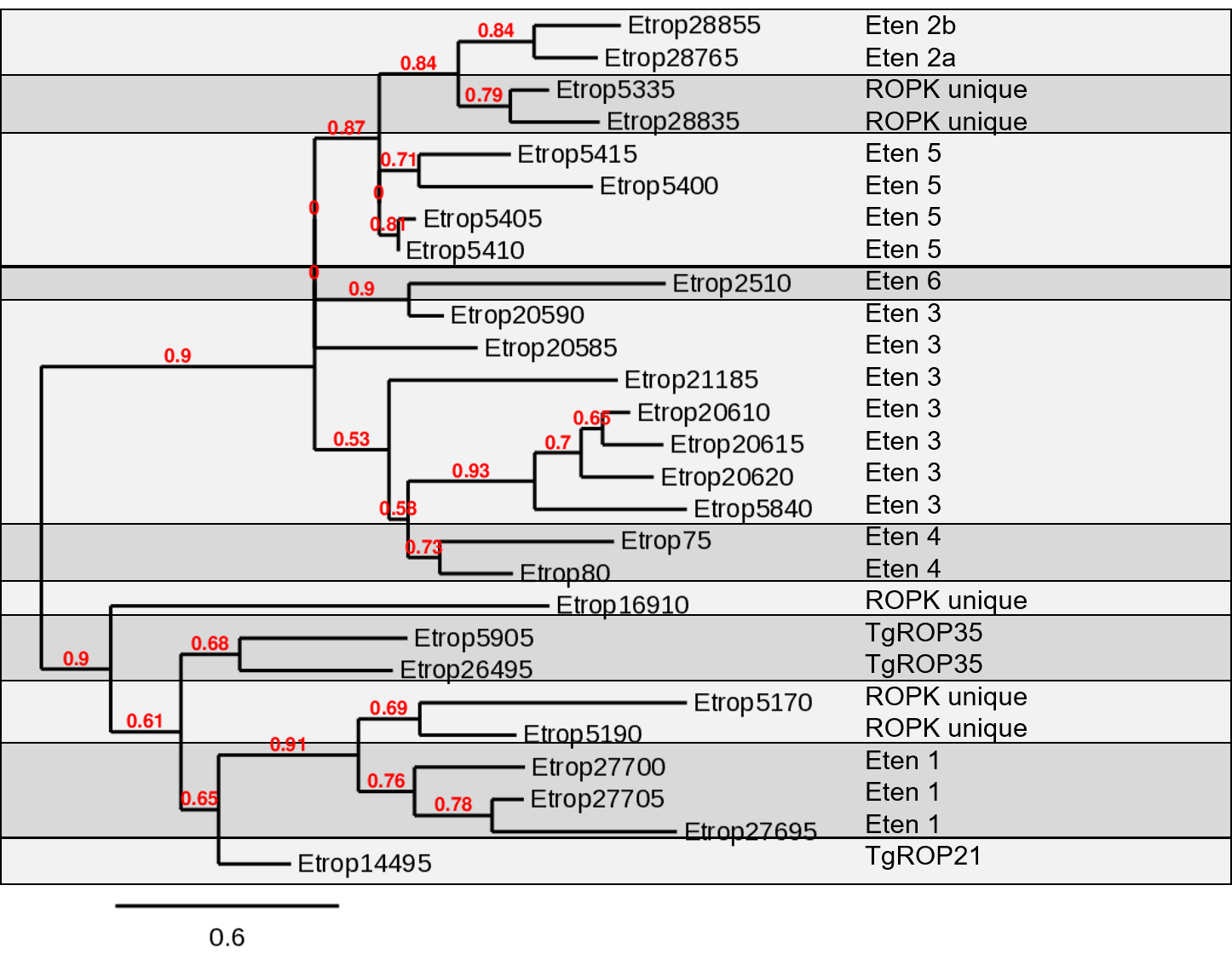

Supplement: Supplementary file 1 [file microorganisms-09-01621-s001.zip › Figure-S1.tif]

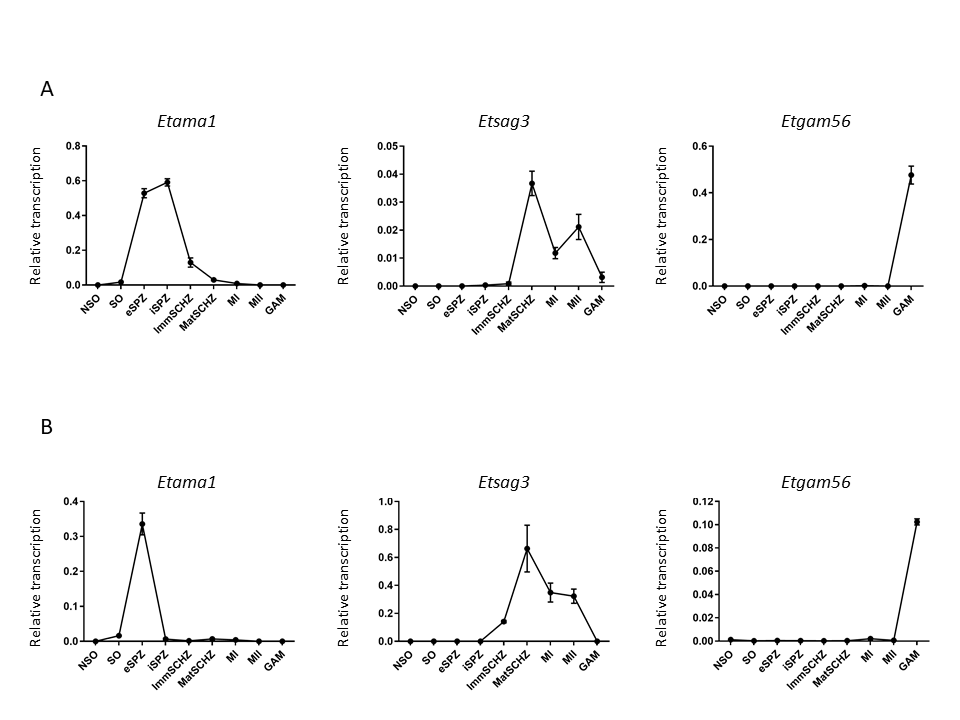

Supplement: Supplementary file 1 [file microorganisms-09-01621-s001.zip › Figure-S2.tif]

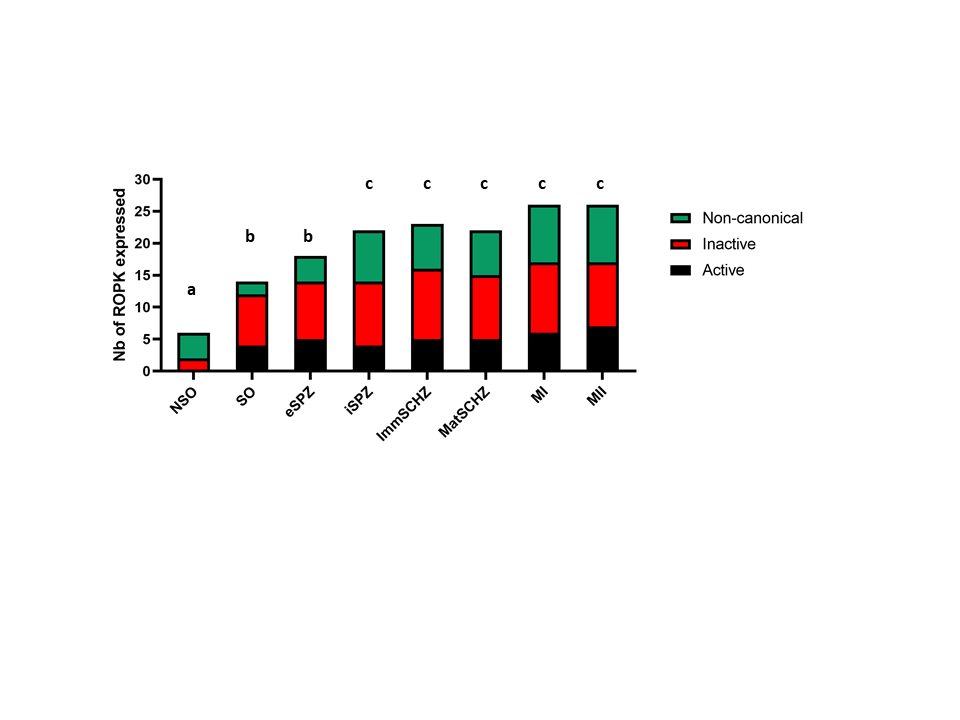

Supplement: Supplementary file 1 [file microorganisms-09-01621-s001.zip › Figure-S3.tif]

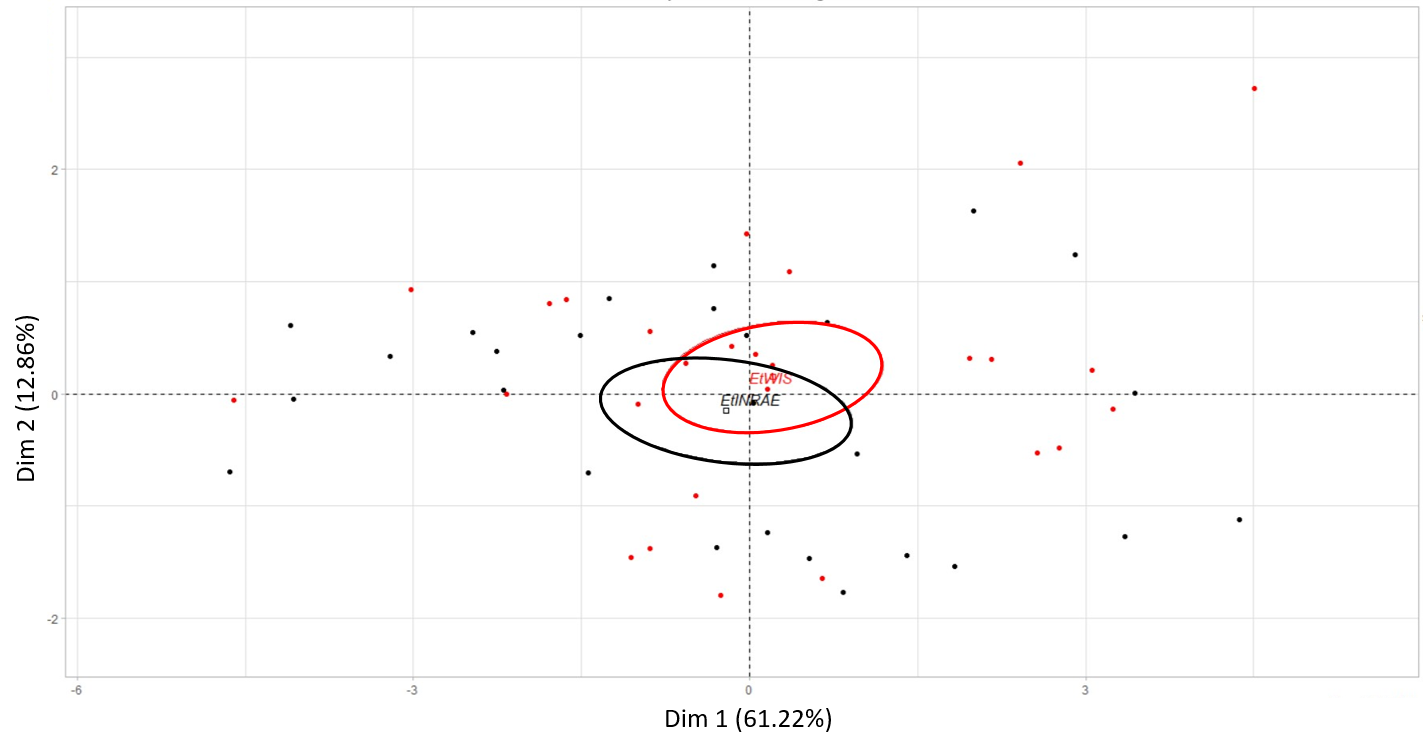

Supplement: Supplementary file 1 [file microorganisms-09-01621-s001.zip › Figure-S4.tif]
